# Efficient Surveillance of *Plasmodium knowlesi* Genetic Subpopulations, Malaysian Borneo, 2000–2018

**DOI:** 10.3201/eid2607.190924

**Published:** 2020-07

**Authors:** Paul C.S Divis, Ting H. Hu, Khamisah A. Kadir, Dayang S.A. Mohammad, King C. Hii, Cyrus Daneshvar, David J. Conway, Balbir Singh

**Affiliations:** Universiti Malaysia Sarawak, Kota Samarahan, Malaysia (P.C.S. Divis, T.H. Hu, K.K. Kadir, D.S.A Mohammad, C. Daneshvar, D.J Conway, B. Singh);; London School of Hygiene and Tropical Medicine, London, UK (P.C.S. Divis, D.J. Conway);; Kapit Hospital, Kapit, Malaysia (K.C. Hii);; University Hospitals Plymouth National Health Service Trust, Plymouth, UK (C. Daneshvar)

**Keywords:** Plasmodium knowlesi, malaria, zoonosis, surveillance, Malaysia, vector-borne infections, mosquitoes, Anopheles, Malaysian Borneo, parasites, parasitic diseases

## Abstract

Population genetic analysis revealed that *Plasmodium knowlesi* infections in Malaysian Borneo are caused by 2 divergent parasites associated with long-tailed (cluster 1) and pig-tailed (cluster 2) macaques. Because the transmission ecology is likely to differ for each macaque species, we developed a simple genotyping PCR to efficiently distinguish between and survey the 2 parasite subpopulations. This assay confirmed differences in the relative proportions in areas of Kapit division of Sarawak state, consistent with multilocus microsatellite analyses. Analyses of 1,204 human infections at Kapit Hospital showed that cluster 1 caused approximately two thirds of cases with no significant temporal changes from 2000 to 2018. We observed an apparent increase in overall numbers in the most recent 2 years studied, driven mainly by increased cluster 1 parasite infections. Continued monitoring of the frequency of different parasite subpopulations and correlation with environmental alterations are necessary to determine whether the epidemiology will change substantially.

The monkey parasite *Plasmodium knowlesi* was discovered to be a common cause of malaria in humans in 2004, initially from investigations in the Kapit division of Sarawak state, Malaysian Borneo (*1*). Humans acquire infection primarily from wild long-tailed (*Macaca fascicularis*) and pig-tailed (*M. nemestrina*) macaque reservoirs (*2*); *Anopheles* mosquitoes of the leucosphyrus group are vectors (*3*,*4*). *P. knowlesi* malaria has been described across Southeast Asia, but most clinical cases are still reported in Malaysian Borneo (*3*,*5*–*8*). In 2017 and 2018, a total of 7,745 cases were reported in Malaysia, 86.8% of which were detected in Malaysian Borneo (B. Singh, unpub. data) (*9*). *P. knowlesi* infections can be asymptomatic (*10*,*11*), and clinical cases exhibit a wide spectrum of disease ranging from mild symptoms to death (*3*).

Population genetic surveys of *P. knowlesi* infections in humans across Malaysia have revealed 2 divergent subpopulations of the parasite in Malaysian Borneo that are associated with the 2 macaque species locally, suggesting 2 independent zoonoses (*12*,*13*). The cluster 1 type has been associated with long-tailed macaques and the cluster 2 type with pig-tailed macaques (*12*). The existence of 2 sympatric subpopulations also has been confirmed by whole-genome sequencing (WGS) of *P. knowlesi* from patients in Malaysian Borneo (*13*,*14*). In peninsular Malaysia on the Asia mainland, all cases have been caused by another subpopulation, cluster 3, that has not been detected in Malaysian Borneo (*13*,*15*). Limited WGS (*14*,*15*) and microsatellite (*13*) genotyping of *P. knowlesi* isolates derived from human and only long-tailed macaque hosts from peninsular Malaysia showed allopatric divergence for this subpopulation cluster from those of Malaysian Borneo because of geographic separation by the South China Sea.

Increasing numbers of *P. knowlesi* malaria cases detected might be due to increased zoonotic exposure along with a reduction of endemic malaria parasite species (*16*). With the recent identification of different zoonotic *P. knowlesi* genetic subpopulations, determining whether these populations vary in frequency over space and time is important. Interactions with vectors and reservoir hosts will affect distributions of these parasite subpopulations, and human contacts with these vectors and reservoir hosts would determine zoonotic incidence. Because the previously used microsatellite genotyping and WGS are time consuming and expensive, we developed a simple PCR to discriminate between the 2 *P. knowlesi* subpopulations in Malaysian Borneo.

## Materials and Methods

### Identification of Cluster-Specific Markers

We identified high-quality bi-allelic single-nucleotide polymorphisms (SNPs) from whole-genome sequence data of *P. knowlesi* isolates from cluster 1 (n = 38) and cluster 2 (n = 10) subpopulations (*15*). We identified 9,293 SNPs with complete fixation of alternative alleles between the 2 subpopulations. Oligonucleotides for allele-specific PCRs were designed on the basis of genome regions with a high density of fixed SNPs and having multiple SNPs <5 nt apart, especially at the 3′ ends of the designed primers. For each subpopulation, we chose 5 pairs of forward and reverse PCR primers to evaluate for genotyping assays (Appendix Table 1).

### Cluster-Specific Genotyping PCR

PCRs to discriminate subpopulation clusters were optimized in 11-μL reaction volume containing 1X Green GoTaq Flexi buffer (Promega, https://www.promega.com), 5 mmol/L MgCl2, 0.275 U GoTaq DNA polymerase, 0.2 mmol/L dNTP (Bioline, https://www.bioline.com), 1–2-μL DNA template, and 0.25 μmol/L each forward and reverse primers. We tested 2 thermal cycling methods to determine the optimum annealing temperature. The first conventional method was 94°C for 2 min, followed by 35 cycles of initial denaturation of 94°C for 30 s, gradient annealing from 50°C to 66°C for 1 min, extension at 72°C for 30 s, and final elongation at 72°C for 2 min. For the second method, we used the touchdown PCR to increase the specificity and sensitivity in amplification (*17*) with the following 2 phases. We initiated the first phase with denaturation at 94°C for 2 min, followed by 10 cycles of touchdown program of 94°C for 30 s, annealing of 10°C above the primer melting temperature for 45 s and 72°C for 30 s, with a decreased on 1°C of the annealing temperature every cycle. Then, we performed an additional 25 cycles as follows: 94°C for 30 s, annealing of 5°C below the primer annealing primer melting temperature for 45 s, 72°C for 30 s, and final elongation at 72°C for 5 min. We analyzed PCR products by electrophoresis on agarose gel stained with ethidium bromide and visualized them under ultraviolet transillumination.

### DNA and Blood Samples from Malaria Infections

To validate allele-specific PCRs, we obtained DNA samples of *P. knowlesi* infections in Malaysian Borneo from previous studies (*12*,*13*,*15*). Samples from humans were from Betong (n = 29), Kanowit (n = 34), Miri (n = 46), Sarikei (n = 23), Kapit (n = 52), Kudat (n = 46), Ranau (n = 62), and Tenom (n = 48); we also used samples from long-tailed macaques (n = 10) and pig-tailed macaques (n = 5) from Kapit (*2*). We determined subpopulation assignments (cluster 1 or cluster 2) of each infection by analyzing multilocus microsatellite data (*12*) or whole-genome sequence data (*15*). *Plasmodium* DNA controls of humans (*P. falciparum*, *P. vivax*, *P. malariae*, and *P. ovale*) and macaques (*P. knowlesi*, *P. inui*, *P. cynomolgi*, *P. fieldi*, and *P. coatneyi*) were also used for specificity tests of the PCRs.

To determine the frequency of the 2 *P. knowlesi* subpopulations over time, we investigated samples derived from Kapit division, Sarawak state, Malaysian Borneo, where high incidence of cases has been reported (*7*). New samples were collected as dried blood spots on filter papers (3MM Whatman, https://www.sigmaaldrich.com) from *P. knowlesi* clinical cases in Kapit Hospital during September 2016–May 2018 (440 cases). DNA was extracted and *P. knowlesi* confirmed by species-specific nested PCRs (*2*) at the Malaria Research Centre, Universiti Malaysia Sarawak (UNIMAS, Kota Samarahan, Malaysia). We also analyzed 764 DNA samples from persons with *P. knowlesi* infection at Kapit Hospital collected previously: March 2000–December 2002, 110 samples (*1*); March 2006–February 2008, 176 samples (*18*); and June 2013–mid-September 2016, 478 samples (*13*).

### Data Analysis and Ethics Approval

We tabulated genotyping data in Excel and conducted statistical analysis using R (https://www.r-project.org). We also explored temporal patterns for overall *P. knowlesi* subpopulation cases over the previous 5 years using the R package seasonal decomposition of time series (STL) by LOESS (locally estimated scatterplot smoothing) (*19*). We generated STL decomposition using periodic as smoothing parameter for the seasonal component (n_s_) with no robustness iterations. LOESS is the seasonal-trend decomposition method used in estimating trend from the time series data. The Medical Ethics Committees of UNIMAS (NC-21.02/03-02 Jld 2 [*19*]) and the Medical Research and Ethics Committee, Malaysian Ministry of Health (NMRR-16-943-31224), approved this study.

## Results

### Development of Simple PCR to Discriminate *P. knowlesi* Cluster 1 and 2 Subpopulations

**Figure 1:**
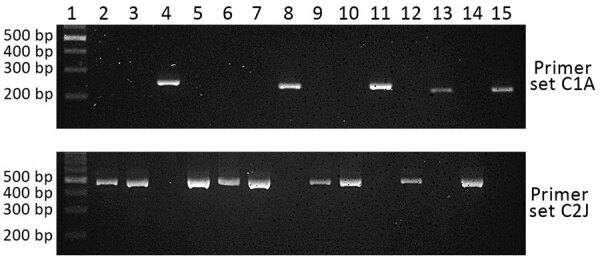
Specificity of PCR primer sets C1A and C2J for discriminating *Plasmodium knowlesi* infections of cluster 1 and cluster 2 subpopulations, Kapit division, Sarawak state, Malaysian Borneo. Lane 1, DNA ladder; lane 2, KT025; lane 3, KT027; lane 4, KT029; lane 5, KT031; lane 6, KT042; lane 7, KT055; lane 8, KT057; lane 9, KT114; lane 10, BTG025; lane 11, BTG026; lane 12, BTG033; lane 13, BTG035; lane 14, BTG044; lane 15, BTG062. Primer sequences and amplification conditions are shown in Appendix Table 1.

We tested 10 PCRs on the basis of fixed SNP differences for performance in discriminating the 2 sympatric *P. knowlesi* subpopulations. Of these, 1 assay each for cluster 1 (primer pair C1A) and cluster 2 (primer pair C2J) showed complete specificity when tested on 5 cluster 1 and 9 cluster 2 DNA samples (Figure 1; Appendix Table 2) that were previously confirmed by microsatellites cluster assignments (*13*). These primers were also *P. knowlesi*–specific and did not show amplification on DNA of other simian malaria parasites (*P. coatneyi*, *P. cynomolgi*, *P. fieldi*, and *P. inui*) or human malaria parasites (*P. falciparum*, *P. vivax*, *P. malariae*, and *P. ovale*), or host DNA from humans or either macaque host species (Appendix Figure 1).

We further tested these primer sets for sensitivity using pure DNA of cluster 1 samples with starting parasitemias of 13,793 parasites/μL blood and cluster 2 samples with starting parasitemias of 8,017 parasites/μL blood. We prepared 5-fold serial dilutions to serve as template in separate PCRs. Tests of sensitivity showed limit of detection at ≈4 parasites/μL blood for primer pair C1A and ≈13 parasites/μL blood for primer pair C2J (Appendix Figure 2).

### Proportions of *P. knowlesi* Subpopulations in Different Areas

**Figure 2:**
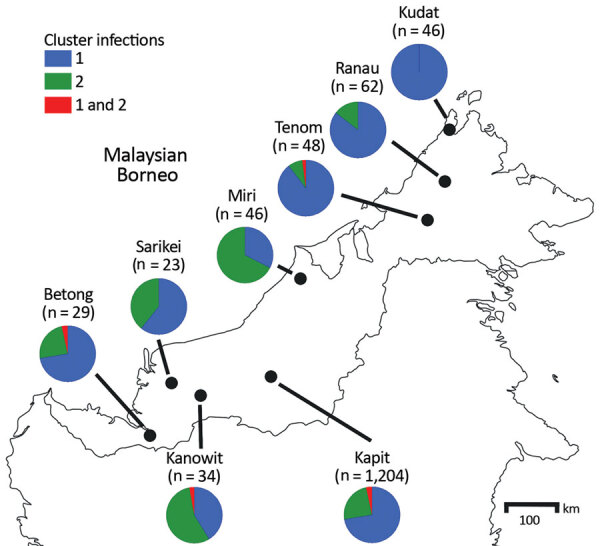
Proportions of cluster 1 and cluster 2 *Plasmodium knowlesi* infections as defined with the cluster-specific PCRs, Malaysian Borneo. A total of 1,492 *P. knowlesi* infections from humans were genotyped using the cluster-specific PCR primers. The exact numbers for each location are shown in Appendix Table 3.

Analysis of DNA samples of 1,492 *P. knowlesi* malaria cases in different regions of Malaysian Borneo showed cluster 1 as the predominant subpopulation (70%), followed by cluster 2 (28%) (Figure 2; Appendix Table 3). Only 2% of infections were positive for both primer sets, and these infections had mixed genotypes as identified by previous microsatellite analysis (*12*,*13*). Cluster 1 infections were more common at most individual locations sampled throughout Malaysian Borneo, except for Miri and Kanowit, where cluster 2 infections were more common.

We also tested the primer sets on 15 natural infections of wild macaques (10 long-tailed and 5 pig-tailed) from Kapit. *P. knowlesi* infections from all macaques showed concordant macaque host association (cluster 1 in long-tailed macaques and cluster 2 in pig-tailed macaques), except for 1 long-tailed macaque that was infected by both cluster 1 and cluster 2 parasites (Appendix Table 3).

### Temporal Analysis of *P. knowlesi* Subpopulation Types in a High-Incidence Area

**Figure 3:**
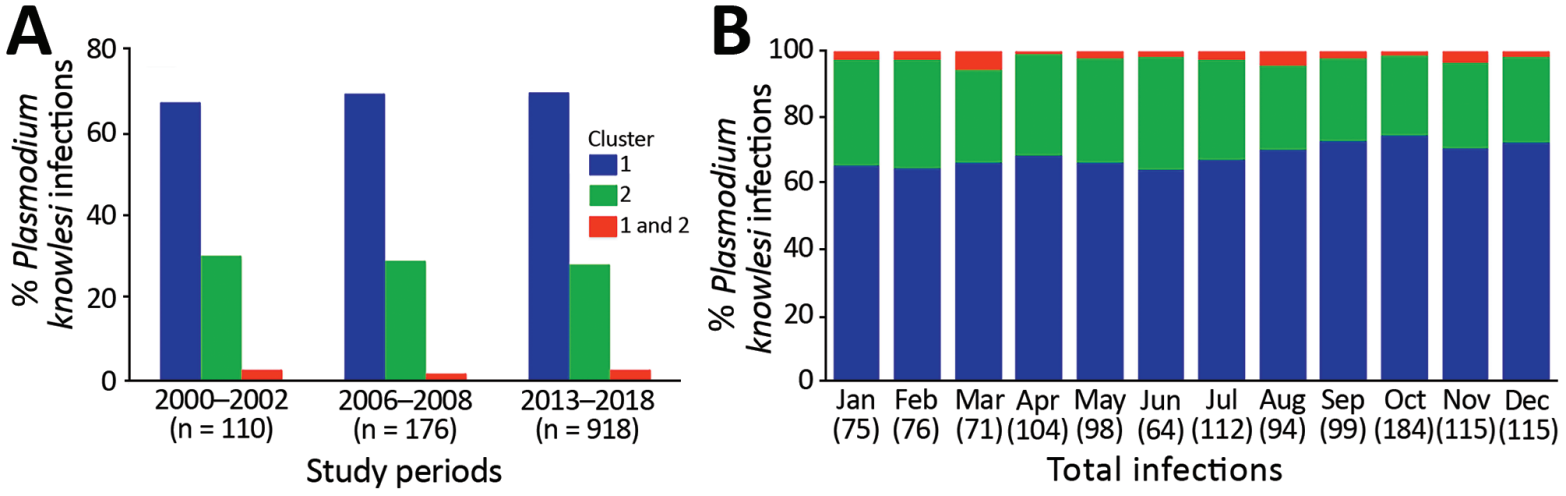
Proportions of *Plasmodium knowlesi* subpopulations in humans of the Kapit division, Sarawak state, Malaysian Borneo, 2000–2018. A total of 1,204 *P. knowlesi* infections were genotyped, and distribution patterns of subpopulation clusters were analyzed for 3 different study periods (A) and by months (B). Numbers in parentheses indicate numbers of cases. Blue indicates cluster 1; green, cluster 2; red, clusters 1 and 2.

In Kapit, where a high incidence of cases has been reported since the zoonosis was discovered to be common (*1*,*7*,*18*), we analyzed DNA samples from 1,204 human *P. knowlesi* infections collected in 3 study periods during 2000–2018. Of those infections, 69% belonged to the cluster 1 subpopulation (Figure 3, panel A; Appendix Table 4). Most (28%) of the remaining *P. knowlesi* infections belonged to the cluster 2 subpopulation, and only 2% had parasites of both types. During 3 study periods of 2000–2002 (n = 110 cases), 2006–2008 (n = 176 cases), and 2013–2018 (n = 918 cases), the proportion of cluster 1 and cluster 2 subpopulations showed similar patterns of distribution with cluster 1 subpopulation as the predominant ones (Pearson p = 0.74). We also observed similar patterns across 12 months of these study periods (Figure 3, panel B), indicating a lack of strong seasonal variation between cluster 1 and cluster 2 infections in Kapit (p = 0.56 by Fisher exact test).

**Figure 4:**
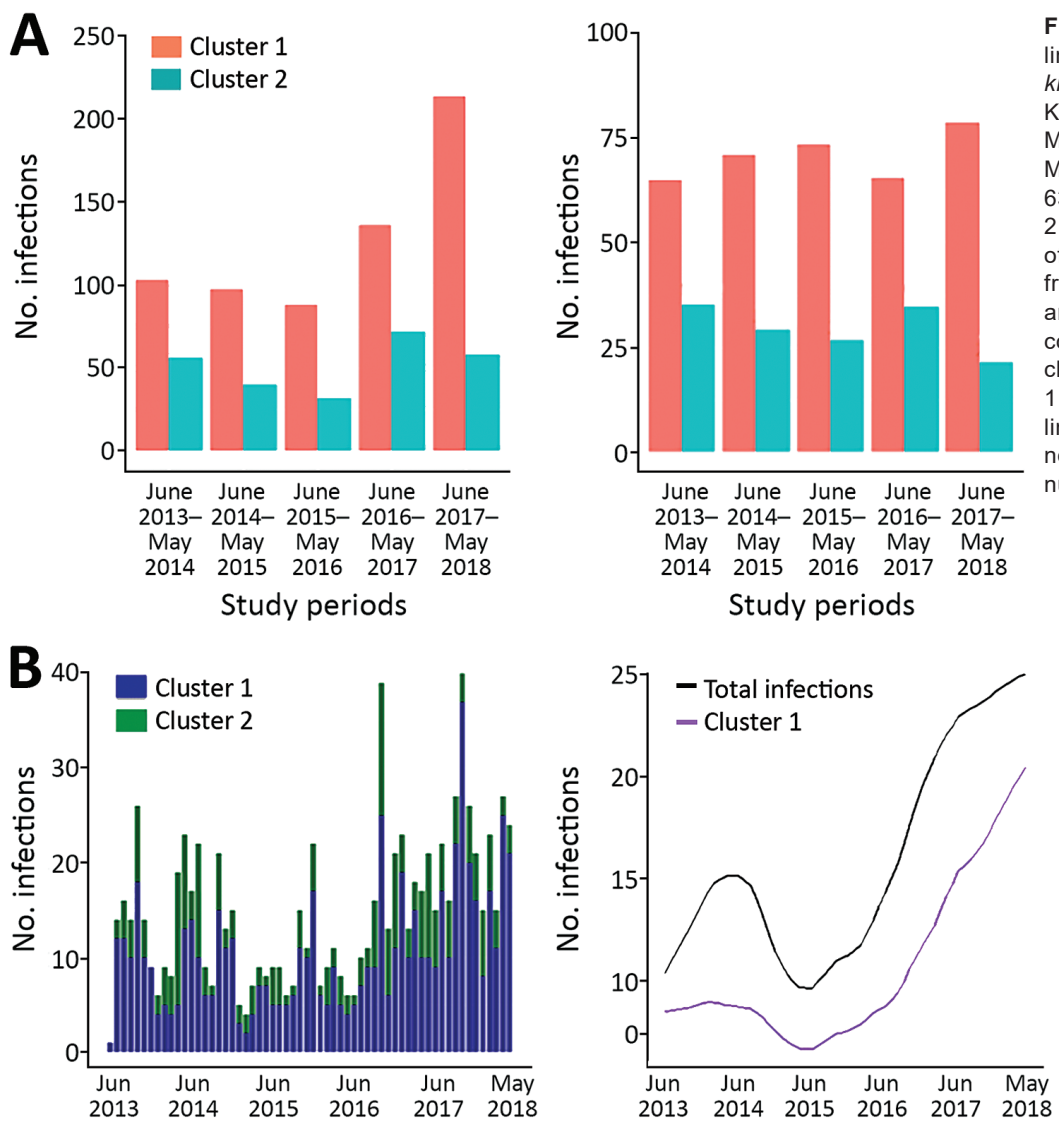
Frequency and trend line patterns of *Plasmodium knowlesi* subpopulations in Kapit division, Sarawak state, Malaysian Borneo, June 2013–May 2018. A) Distribution of 637 cluster 1 and 258 cluster 2 infections during each year of the 5-year period. B) The frequency pattern of infections and estimation of trend for the combination of cluster 1 and cluster 2 infections, and cluster 1 infection alone. The trend line of cluster 2 infections is not shown because of a low number of cases.

We conducted a more intensive analysis on infections sampled during the most recent 5-year study period (June 2013–May 2018); we made a particular effort to recruit most of the case-patients seeking care at Kapit Hospital during this time. Of the 918 infections genotyped from this period, 637 were cluster 1 infections, 258 were cluster 2 infections, and 23 were mixed cluster infections. The proportion of cluster 1 infections always ranged from 63% to 78% (Pearson p = 0.007) and was highest in the most recent year. The total number of *P. knowlesi* cases was also highest in the most recent year (Figure 4, panel A). Using an STL decomposition method of analysis based on numbers plotted on a monthly basis (Figure 4, panel B), we noted a trend of increasing numbers of cases overall, as well as of the cluster 1 subpopulation separately, from June 2016 onward. We found no significant trend for cluster 2 cases separately.

## Discussion

On the basis of fixed SNP differences between the 2 *P. knowlesi* genetic subpopulations in Malaysian Borneo, identified by whole-genome sequence data analysis (*14*,*15*), we successfully developed an allele-specific PCR for discriminating these in large-scale studies. This method enables identification of cluster 1 and cluster 2 subpopulations from field isolates throughout the sympatric distribution, without the need to perform population genetic analysis of multilocus data on all samples. A different *P. knowlesi* subpopulation termed cluster 3 has been described to account for all cases in Peninsular Malaysia (*13*,*15*), but we did not study that subpopulation because it has not been detected in Malaysian Borneo.

The use of these allele-specific PCRs is limited to *P. knowlesi* infections in Malaysian Borneo because they were designed on the basis of the genome sequences of the cluster 1 and cluster 2 parasites found there. These assays could potentially be applied to analyze *P. knowlesi* infections in Kalimantan, Indonesian Borneo, which shares international borders with Malaysian Borneo. The allopatric divergence of cluster 3 subpopulations in Peninsular Malaysia (*13*) indicates the need for development of a separate allele-specific PCR for those parasites, which would require analysis of whole-genome data from substantially more *P. knowlesi* isolates from Peninsular Malaysia.

The validation of this allele-specific PCR shows remarkable sensitivity with single-round (nonnested) amplification, even at a parasitemia of ≈4 parasites/μL blood. Although all samples tested from patients at Kapit Hospital could be genotyped, this PCR would have limitations on subpatent *P. knowlesi* infections when parasites are undetectable under the microscope (*10*,*11*,*20*).

No clear evidence of direct human–mosquito–human transmission of *P. knowlesi* has yet been detremined, although such transmission might be occurring. Most hospitalized *P. knowlesi* malaria patients have been adult farmers or logging camp workers, who regularly spend time in forests or forest fringes, or travelers entering forests (*3*), suggesting that macaque–vector–human transmission is the primary route of infection (*21*). Although the distribution of long-tailed and pig-tailed macaques overlaps throughout Southeast Asia, these macaque species show different habitat preferences (*22*). The preference of long-tailed macaques for cropland, wetland, and urban areas brings them in close proximity to humans, but whether transmission occurs outside of the forests is not known. The widespread distribution of long-tailed macaques corresponds with the cluster 1 parasite subpopulation accounting for most *P. knowlesi* infections in Kapit. The lower frequency of the cluster 2 subpopulation in humans is as expected, given that those parasites are associated with pig-tailed macaques, which occur in more remote forested areas (*22*).

Like many other vectorborne parasitic diseases, malaria is sensitive to environmental changes such as deforestation (*23*); the association between incidences of *P. knowlesi* infections and environmental changes in Sabah, Malaysian Borneo, supports this fact (*16*,*24*), because overall numbers of cases have increased in the past few years (*8*). We have shown that most of the cases in Sabah are of the cluster 1 genetic subpopulation of *P. knowlesi*, and that numbers of this parasite subpopulation have increased in Kapit in the most recent 2 years of the study period. Whether changes in the landscape in Sarawak or other factors have contributed to the increasing numbers of *P. knowlesi* cases needs to be studied. Determining whether any significant differences exist between the clinical outcomes after infection with the different subpopulations also is important. Such determination requires collecting detailed clinical and laboratory data on *P. knowlesi* patients infected with the 2 different subpopulation clusters and performing careful association study analysis to deliver robust inference for the benefits of both clinicians and public health experts.

*P. knowlesi* infections require separate efforts from those being targeting elimination of human malaria (*P. falciparum* and *P. vivax*), particularly in Malaysia, where zoonotic malaria is dominant (*25*). Although *P. knowlesi* is not part of the national elimination program (*26*), monitoring this parasitic infection is crucial because of its increasing incidence. Zoonotic malaria requires new strategies in prevention and control, including monitoring of the different parasite genetic subpopulations.

Appendix Additional results for *Plasmodium knowlesi* genetic subpopulations, Malaysian Borneo.
