# Supplementary material for: Efficient Surveillance of Plasmodium knowlesi Genetic Subpopulations, Malaysian Borneo, 2000–2018
Source: Emerg Infect Dis. 2020 Jul;26(7):1392–8. doi: 10.3201/eid2607.190924 (PMC7323547; doi:10.3201/eid2607.190924)
Supplement: Appendix — Additional results for Plasmodium knowlesi genetic subpopulations, Malaysian Borneo. [file 19-0924-Techapp-s1.pdf]

Article DOI: https://doi.org/10.3201/eid2607.190924

Efficient Surveillance of *Plasmodium knowlesi* Genetic Subpopulations, Malaysian Borneo, 2000–2018

Appendix

Appendix Table 1. Unique primer pairs designed at each locus to discriminate the 2 *Plasmodium knowlesi* subpopulation clusters

Subpopulation, set	Chromosome, location of locus*	Primer ID	Sequence, 5′→3′†
Cluster 1			
C1A	1	C101AF	GTTTGGTACGTTCAAGTGTTGC**G**CTA**TGG**
	(745,563–745,763)[a]	CX01AR	CGTCTTCCGCTTGTCGTTTTCCATGTAC
C1C	6	C106BF	GATATAACCACATGTTTGCTTCGAAGGAA
	(17,264–17,523)[c]	C106BR	GGAAAGGTACCTCTTCCTCATAGTCC**C**
C1B	6	C106AF	TCCATGTGCACCCTGGCATACATGG**T**A**C**
	(35,925–36,162)[b]	C106AR	TGTACAGAGTGTACAGGAGCTGGGAC
C1D	6	C106CF	GGATGATTTAGGTAAGGATGAGGAG**G**G**T**
	(1,014,117 – 1,014,286)[d]	CX06CR	CGTCATCCTTATCCTTTTTACCCTTATCC
C1E	6	C106DF	GATGATAATTATCTTAAAGAGCCGGA**TG**
	(1,039,470–1,039,954)[e]	C106DR	CAAGACATTATGAACATTGGACCGA**T**T**A**
Cluster 2			
C2F	1	C201AF	GTTTGGTACGTTCAAGTGTTGC**T**CTA**CAT**
	(745,563–745,763)[a]	CX01AR	CGTCTTCCGCTTGTCGTTTTCCATGTAC
C2G	6	C206AF	GATATAACCACATGTTTGCTTCGAA**A**GA**G**
	(17,264 – 17,523)[c]	C206AR	GGAAAGGTACCTCTTCCTCATAGTCC**A**
C2H	6	C206BF	TCCATGTGCACCCTGGCATACATGG**C**A**T**
	(35,925–36,162)[b]	C106AR	TGTACAGAGTGTACAGGAGCTGGGAC
C2I	6	C206CF	GGATGATTTAGGTAAGGATGAGGAG**T**G**C**
	(1,014,117–1,014,286)[d]	CX06CR	CGTCATCCTTATCCTTTTTACCCTTATCC
C2J	6	C206DF	GATGATAATTATCTTAAAGAGCCGGA**G**
	(1,039,470–1,039,954)[e]	C206DR	CAAGACATTATGAACATTGGACCGA**C**T**G**

*Superscripted letters at the end of locations indicate same loci for both subpopulation clusters.
†Single-nucleotide polymorphisms with complete fixed (FST = 1) as shown at the 3′-end sequence are in bold and underlined.

Appendix Table 2. Summary of PCR optimizations for discriminating 2 subpopulations of *Plasmodium knowlesi* infection*

Primer set	Types of PCR	Optimum annealing temperature, °C	Allele specificity	PCR summary
C1A	Conventional PCR	60	Cluster 1	Cluster 1-specific
C1B	Conventional PCR	ND	ND	Weak amplification
C1C	Conventional PCR	61	Cluster 1 and Cluster 2	Not specific
C1D	Conventional PCR	ND	ND	Weak amplification
C1E	Touchdown PCR	68, 60	Cluster 1 and Cluster 2	Not specific
C2F	Conventional PCR	60	Cluster 2	Cluster 1–specific but inconsistent
C2G	Touchdown PCR	68, 60	Cluster 1 and Cluster 2	Not specific
C2H	Conventional PCR	ND	ND	Weak amplification
C2I	Conventional PCR	ND	ND	Weak amplification
C2J	Touchdown PCR	68, 62	Cluster 2	Cluster 2–specific

*The specificity of primers tested on DNA of Cluster 1 and Cluster 2 clinical infections where cluster identity of infections was confirmed previously by multilocus microsatellite analysis or whole-genome sequencing. ND, further optimization of annealing temperature was not done due to weak amplification during gradient PCR when visualized on agarose gel.

Appendix Table 3: Specificity of allele-specific PCRs for primer sets C1A and C2J for *Plasmodium knowlesi* Cluster 1 and Cluster 2 subpopulations, respectively, Malaysian Borneo*

			Infections per primer set, no. (%)		
Location	Host	Total	C1A	C2J	Both
Betong	Human	29	21 (72)	7 (24)	1 (4)
Kanowit	Human	34	14 (41)	19 (56)	1 (3)
Miri	Human	46	15 (33)	31 (67)	0
Sarikei	Human	23	14 (61)	9 (39)	0
Kapit	Human	1,204	833 (69)	342 (29)	29 (2)
Kudat	Human	46	46 (100)	0	0
Ranau	Human	62	53 (85)	9 (15)	0
Tenom	Human	48	43 (90)	4 (8)	1 (2)
Kapit	Long-tailed macaque	10	9 (90)	0	1 (10)
Kapit	Pig-tailed macaque	5	0	5 (100)	0

*All DNA samples were blinded with regards to their cluster assignments deduced from microsatellite (*1*) and whole genome sequencing (*2*) analyses.

Appendix Table 4. *Plasmodium knowlesi* subpopulation genotyping results for 3 study periods, Kapit division, Sarawak state, Malaysian Borneo, 2000–2018

		Infections, no. (%)		
Study period	Total no. samples	Cluster 1	Cluster 2	Mixed
2000–2002	110	74 (67)	33 (30)	3 (3)
2006–2008	176	122 (69)	51 (29)	3 (2)
2013–2018	918	637 (69)	258 (28)	23 (3)
Total	1,204	833 (69)	342 (28)	29 (3)

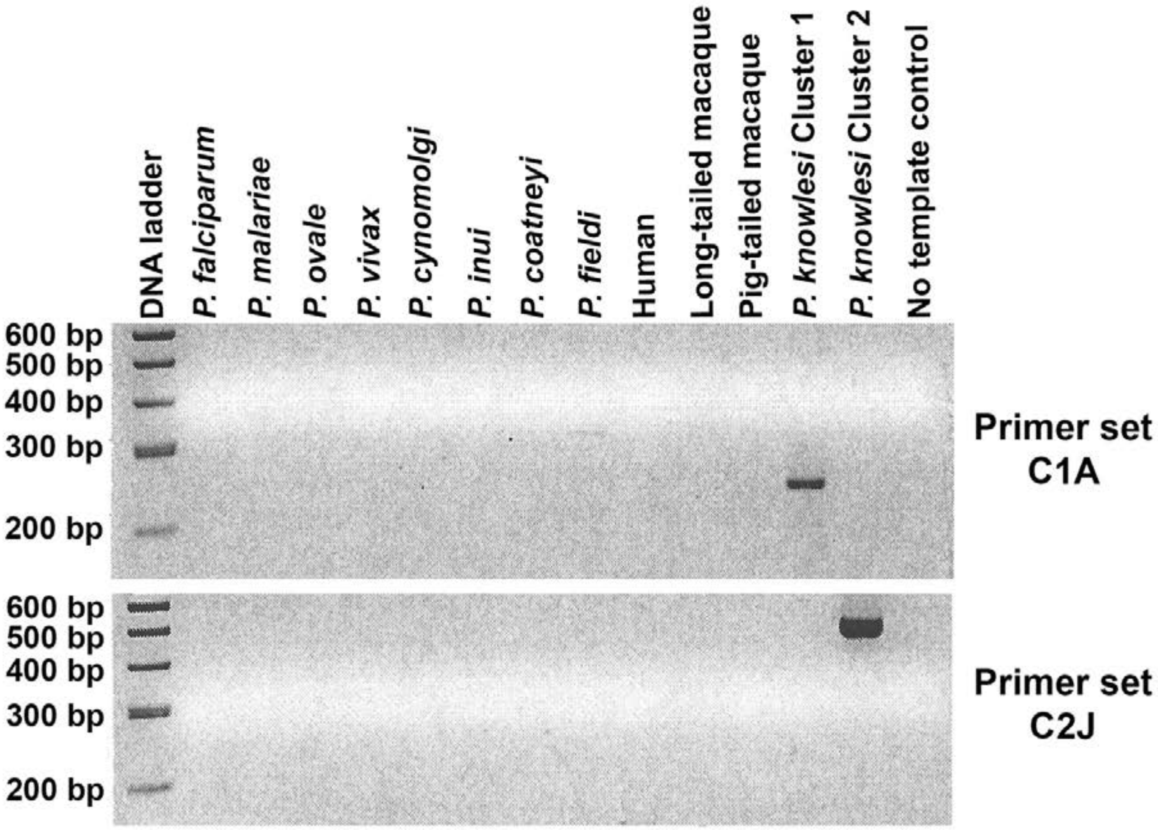

Appendix Figure 1. Species specificity of PCR primer sets C1A and C2J for discriminating *Plasmodium knowlesi* infections of Cluster 1 and Cluster 2 subpopulations. Gel electrophoresis on 2.7% agarose shows both primer sets did not cross-react to DNA of human *Plasmodium* species (*P. falciparum*, *P. malariae*, *P. ovale*, and *P. vivax)* and common *Plasmodium* species found in Southeast Asian macaques *(P. cynomolgi*, *P. inui*, *P. coatneyi*, and *P. fieldi*), as well as DNA of humans, long-tailed macaque, and pig-tailed macaque.

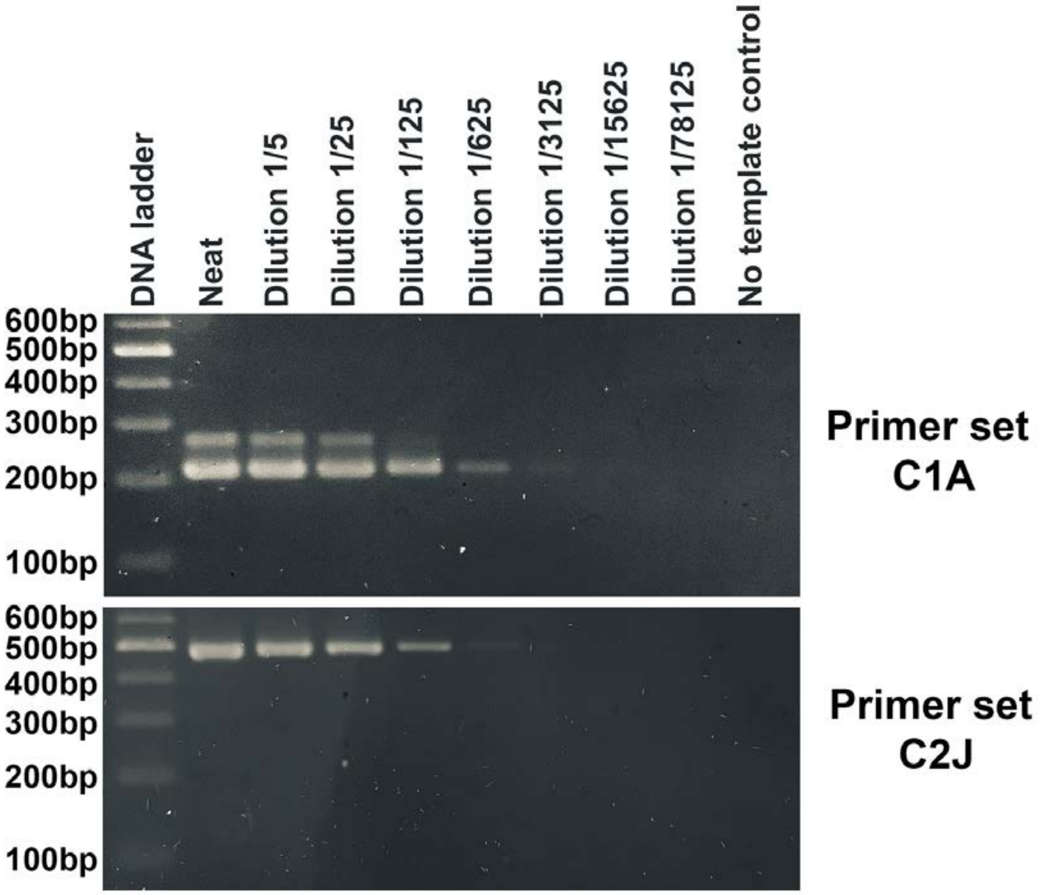

Appendix Figure 2. Analytical sensitivity of PCR primer sets C1A and C2J for discriminating *Plasmodium knowlesi* infections of Cluster 1 and Cluster 2 subpopulations. Pure DNA of Cluster 1 sample with parasitemia of 13,793 parasites/μL blood and Cluster 2 sample of 8,017 parasites/μL blood were used (denoted as 'Neat') for respective primer sets C1A and C2J. Each pure DNA sample was diluted 5-fold with nuclease free water and served as template in separate PCRs.

References

1. Divis PC, Singh B, Anderios F, Hisam S, Matusop A, Kocken CH, et al. Admixture in humans of two divergent *Plasmodium knowlesi* populations associated with different macaque host species. PLoS Pathog. 2015;11:e1004888. PubMed https://doi.org/10.1371/journal.ppat.1004888

2. Assefa S, Lim C, Preston MD, Duffy CW, Nair MB, Adroub SA, et al. Population genomic structure and adaptation in the zoonotic malaria parasite *Plasmodium knowlesi*. Proc Natl Acad Sci U S A. 2015;112:13027–32. PubMed https://doi.org/10.1073/pnas.1509534112
